# Investigating the Symptom Presentation of Depression in Children With ADHD

**DOI:** 10.1177/10870547251366783

**Published:** 2025-10-02

**Authors:** Gareth Williams, Victoria Powell, Olga Eyre, Anita Thapar, Lucy Riglin

**Affiliations:** 1 Wolfson Centre for Young People’s Mental Health, Division of Psychological Medicine and Clinical Neurosciences, Cardiff University, Cardiff, UK

**Keywords:** ADHD, depression, symptom presentation, latent profile analysis

## Abstract

**Objective:** ADHD is commonly comorbid with depression and this comorbidity is associated with increased symptom severity and worse outcomes than either condition alone. Depression is highly heterogeneous and may present differently in populations with ADHD. This study aimed to explore different symptom presentations of depression and associated clinical correlates in a clinical ADHD sample. **Method:** We analysed data from the Study of ADHD Genes and Environment (SAGE). Parents completed semi-structured interviews about their child’s psychopathology at baseline (*M*_age_ = 10.9 years) and the Mood and Feelings Questionnaire to capture their child’s depression symptoms approximately 5 years later (*M*_age_ = 14.6 years, *N* = 246). Depression symptom presentations were derived by latent profile analysis. **Results:** Analyses found three presentations of depression symptoms: a ‘low symptoms’ class (48.5% of the sample), a ‘high symptoms’ class (15.5%) with consistently high depression symptoms, particularly for suicidality and poor self-esteem items, and an ‘irritable/poor sleep’ class (36.1%) with high scores for irritability and poor sleep and intermediate levels of other depression symptoms. All three classes had elevated irritability and symptoms that overlap with ADHD. Behavioural problems (oppositional defiant disorder; conduct disorder) were associated with an increased likelihood of being in the high symptoms compared to low symptoms class, and higher autism symptoms were associated with being in the ‘irritable/poor sleep’ compared to low symptoms class. **Conculsion:** Our findings suggest that while young people with ADHD often have elevated depression symptoms, there is notable heterogeneity. Young people with ADHD and behavioural disorders may be particularly at risk of a more severe depression symptom presentations characterised by high suicidal cognitions, whilst those with ADHD and autistic traits may present with more irritability and poor sleep.

## Introduction

Attention Deficit Hyperactivity Disorder (ADHD) is a neurodevelopmental disorder typically originating in childhood, characterised by symptoms of hyperactivity, impulsivity, and inattention (Thapar & Cooper, 2016). With a prevalence of 3.4% in children and adolescents (Polanczyk et al., 2015), ADHD is one of the most prevalent disorders in childhood and a common reason for referral to Child and Adolescent Mental Health Services (CAMHS; Lynch et al., 2023). ADHD can lead to significant impairment that often persists into adulthood and is often comorbid with other neurodevelopmental disorders and also psychiatric disorders (Thapar & Cooper, 2016). Research has consistently shown that individuals with ADHD are at an increased risk of depression (Thapar et al., 2023).

Depression, a mood disorder characterised by persistent feelings of sadness and loss of interest, is the leading mental health contributor to the Global Burden of Disease (Thapar et al., 2022; Vos et al., 2017). Depression exhibits considerable heterogeneity in various respects, such as age of onset, chronicity, recurrence, and associated impairment (Kendler et al., 1996). Young people with ADHD exhibit a 5-fold increased risk of developing depressive disorders compared to the general population (Angold et al., 1999), rising to 6.5-fold within the first year of ADHD diagnosis (Gundel et al., 2018). Evidence suggests that depression can be more severe when comorbid with ADHD, including an earlier onset and longer duration of depressive symptoms, along with a higher suicide rate compared to those without ADHD (Biederman et al., 2008). Depression symptoms may mediate the association between ADHD and increased suicide risk (Balazs et al., 2014). Early recognition of depression is, therefore, essential in this high-risk population.

Depression is heterogeneous not only in terms of disorder onset, course and associated impairment, but it is also heterogeneous in its symptom presentation (Fried & Nesse, 2015; Kendler et al., 1996). This is partly due to its polythetic definition, requiring a minimum number of criteria from a list. One study found over a 1000 unique depression symptom profiles in a sample of depressed patients, demonstrating the substantial variation that exists in depression symptom presentation. However, how symptoms present together is not random; some symptoms cluster together more often than others (Lynall & McIntosh, 2023). Uncertainty exists about whether depression presents with different symptoms in young people with ADHD compared to the general population. A study by Fraser et al. found that while all symptoms of depression were more common in young people with ADHD compared to the general population, there were no differences in which specific depression symptoms were most or least common between the two groups (Fraser et al., 2018). A study by Diler et al. (2007) found evidence to suggest that certain depression symptoms may be better at identifying major depressive disorder (MDD) in young people with ADHD. In this study, the strongest indicators of MDD in young people with ADHD included social withdrawal, anhedonia, depressive cognitions, suicidal thoughts, and psychomotor retardation. Other symptoms, such as mild irritability, miserable/unhappy moods, and symptoms related to sleep, appetite, energy levels, and concentration were not specific to MDD in this sample of young people with ADHD (Diler et al., 2007). There is less clarity on how depression symptoms might cluster together and whether distinct depression symptom profiles exist in young people with ADHD.

Investigating depression symptom profiles can allow us to understand symptom variations across diverse populations, potentially reducing under-diagnosis. Symptom profiles or clusters are composed of stable groups of symptoms that commonly co-occur, are independent of other clusters, and may reveal underlying dimensions of these symptoms (Kim et al., 2005). Depression symptom clusters may include *cognitive* (feelings of worthlessness, guilt, concentration difficulties, and suicidality), *affective* (anhedonia, persistent sadness, and lack of enjoyment), *vegetative* (changes in appetite, weight changes, fatigue, and sleep disturbances), and *somatic* (headaches and unexplained physical pain; Diler et al., 2007; Rice, Riglin, Lomax, et al., 2019). For example, a study by Rice, Riglin, Lomax et al. (2019) comparing symptom profiles of adolescents and adults highlighted heterogeneity in the presentation of depression according to developmental stage, whereby children were more likely to present with vegetative symptoms, whereas adults were more likely to present with anhedonia and concentration problems.

Understanding how depression symptoms might cluster into distinct symptom profiles in those with ADHD can provide valuable insights into the aetiology and presentation of depression in this population. Investigating correlates of depression symptom profiles identified could provide insight into how individuals with distinct characteristics, comorbidities, or risk factors might manifest depression symptoms. A more nuanced understanding of the presentation of depression in young people with ADHD would be beneficial to clinicians in assessing and treating depression in this group. This could facilitate early recognition and intervention for depression – a key research priority according to the World Health Organisation (WHO, 2021). It could also allow clinicians to tailor interventions more effectively. For example, certain groups may benefit from earlier or more intensive interventions, while others may require different treatment approaches (Lynall & McIntosh, 2023).

In this paper, we will examine whether distinct depression symptom profiles exist in a large sample of young people with ADHD, and whether different depression symptom profiles demonstrate different clinical and demographic correlates. Correlates examined will include ADHD subtype (inattentive, hyperactive-impulsive, and combined), ADHD-related impairment, IQ, socioeconomic status, family history of depression, and comorbidities, specifically conduct disorder (CD), oppositional defiant disorder (ODD), and autism spectrum disorder (ASD) and anxiety, all of which are established risk factors for depression with evidence that they may influence depression presentation (Antshel et al., 2009; Capps et al., 2023; Elovainio et al., 2020; Faraone et al., 1998; Graetz et al., 2001; Hirschfeld, 2001; Jensen & Steinhausen, 2015; Mayer et al., 2021; Rice, Riglin, Thapar, et al., 2019; Sprafkin et al., 2007).

## Method

### Sample

This study draws participants from the Study of ADHD Genes and Environment (SAGE), comprising 696 children aged 6 to 18 (*M*_age_ = 10.9, *SD* = 2.99) referred to the study by clinicians from Child and Adolescent Mental Health Services (CAMHS) and paediatric outpatient clinics across South Wales between 2007 and 2011. All children were of British Caucasian origin, 84% were male, and exhibited a mean IQ of 83 (*SD* = 13.35), including 84 participants with an IQ of <70. Participants were excluded from the study if diagnosed with any significant comorbid neurological/neuropsychiatric disorder or genetic syndrome (including fragile X syndrome, tuberous sclerosis, epilepsy, psychosis, Tourette’s syndrome, and any known diagnosis of autism or other pervasive developmental disorder, in line with DSM criteria at that time; Fraser et al., 2018).

Participants received their ADHD diagnosis using the DSM-IV or DSM-III-R criteria, therefore excluding individuals from an ADHD diagnosis if autism spectrum disorder (ASD) was also present. This exclusion criterion was removed in the updated DSM-5 that is used today (Eyre et al., 2017), which is notable because ASD and ADHD are often comorbid (Jensen & Steinhausen, 2015). As SAGE participants were recruited before the release of the DSM-5, no participants with a clinical diagnosis of ASD were included in this sample.

A subsample of participants who were aged 12 years or below at the time of initial data collection was invited to take part in a follow-up study in 2014 to 2015 (on average, 5.4 years after initial participation). Of the 434 eligible families who were sent a follow-up questionnaire, it was completed by 249 families. Full details of the study can be found elsewhere (Eyre et al., 2019).

### Measures

Depression data were collected at follow-up, all other measures were collected at baseline.

#### Mood and Feelings Questionnaire (MFQ)

The Mood and Feelings Questionnaire (MFQ) was used to measure depression symptoms in adolescence at the follow up. The MFQ is a widely used screening instrument for depression, consisting of 34 items each scored on a 0 to 2 scale (0 = ‘not true’, 1 = ‘sometimes true’, 2 = ‘true’; Costello & Angold, 1988). The MFQ has demonstrated validity, internal consistency, diagnostic accuracy, and sensitivity to change as a measure of depression symptoms in young people (Thabrew et al., 2018). Findings from Fraser et al. (2018) suggest that children with ADHD may under-report their depressive symptoms compared to their parents’ assessments. For this reason, the parent reported MFQ (pMFQ) scores were used to ensure depressive symptoms were fully captured. Mean pMFQ scores for each item were interpreted as either ‘low’ (<0.5), ‘slightly elevated’ (0.5–1.0), ‘somewhat high’ (1.0–1.5), or ‘high’ (>1.5), reflecting the options on the MFQ.

#### Child Psychopathology

Parents completed the Child and Adolescent Psychiatric Assessment (CAPA) at baseline. The CAPA is a valid and reliable semi-structured diagnostic interview that asks about symptoms of a range of psychiatric disorders that had been present in the preceding 3 months (Angold & Costello, 2000). Completion of the CAPA allowed interviewers to make DSM-IV research diagnoses. The CAPA data was used alongside teacher reports to confirm the diagnosis of ADHD. It was also used to identify other comorbid psychiatric disorders and their symptoms, including depressive disorders, common childhood anxiety disorders (generalised anxiety disorder and separation anxiety disorder), conduct disorder (CD), and oppositional defiant disorder (ODD), and to derive ADHD impairment score. Interviewers were all trained to a high level of reliability, and all interviews were recorded (Eyre et al., 2019).

#### Autism Screening Score

ASD traits were measured using the Social Communication Questionnaire (SCQ; formerly known as the Autism Screening Questionnaire, ASQ). The SCQ is a 40-item parent-rated questionnaire based on the Autism Diagnostic Interview-Revised (ADI-R). For each item, a score of ‘yes’ (1) or ‘no’ (2) is given for the ASD symptom, giving a maximum score of 40 (Snow, 2013). SCQ scores were analysed as a continuous measure. The SCQ has been validated for autistic traits in the general population, but it is not clear whether this holds true for autistic traits in ADHD (Cooper et al., 2014).

#### Parent and Family History of Depression

Parents were also asked about their family history of depressive disorders, including a lifetime history of depression in the parents, as well as in any first- and second-degree relatives of the child. If first-degree relatives had a diagnosis of depression, a score of 1 was given. For second-degree relatives, a score of 0.5 was given. From this, a total family history score was calculated.

#### Demographic Information and IQ

Parents completed questionnaires at baseline collecting demographic information, including child age and gender, and estimated gross yearly family income. Low income was defined as a gross yearly family income of less than £20,000 per year. The Wechsler Intelligence Scale for Children (WISC-IV) measured child IQ (O’Donnell, 2009). The WISC-IV is a valid and reliable test that is widely used by clinicians (Gygi et al., 2017).

### Statistical Analysis

Heterogeneity in the presentation of depression symptoms was investigated using latent profile analysis (LPA) in Mplus version 8.11. LPA is a person-centred approach that aims to group similar individuals into categories (‘classes’; Muthén & Muthén, 2000). Starting with a single k-class solution, k+1 solutions were fitted until the optimum solution was reached: this was selected based on examination of the loglikelihood, sample size adjusted Bayesian information criterion, Lo–Mendell–Rubin test and bootstrapped likelihood ratio test. Models were fitted using maximum likelihood parameter estimates with standard errors that are robust to the non-independence of these observations (MLR) and full information maximum likelihood (FIML).

Associations between classes and the clinical and demographic correlates were tested using the bias-corrected three-step approach (R3step) in Mplus (Asparouhov & Muthén, 2014); multinomial odds ratios are reported. The lowest depression symptoms class was used as the reference class. As traditional methods of correcting for multiple testing, such as the Bonferroni method, would be too conservative for our study, we used a false discovery rate (FDR) corrected p-value, using R (version 4.2.1; Benjamini et al., 2001; Benjamini & Hochberg, 1995).

## Results

Parent-rated MFQ (pMFQ) data were available for 246 of the 696 participants in the SAGE sample. The sample consisted of 198 (81.5%) male and 45 (18.5%) female participants. The mean pMFQ for the overall sample was 24.43 (*SD* = 15.37) out of a maximum score of 68.

### Description of Depression Symptom Clusters in Children With ADHD

**Figure 1.**
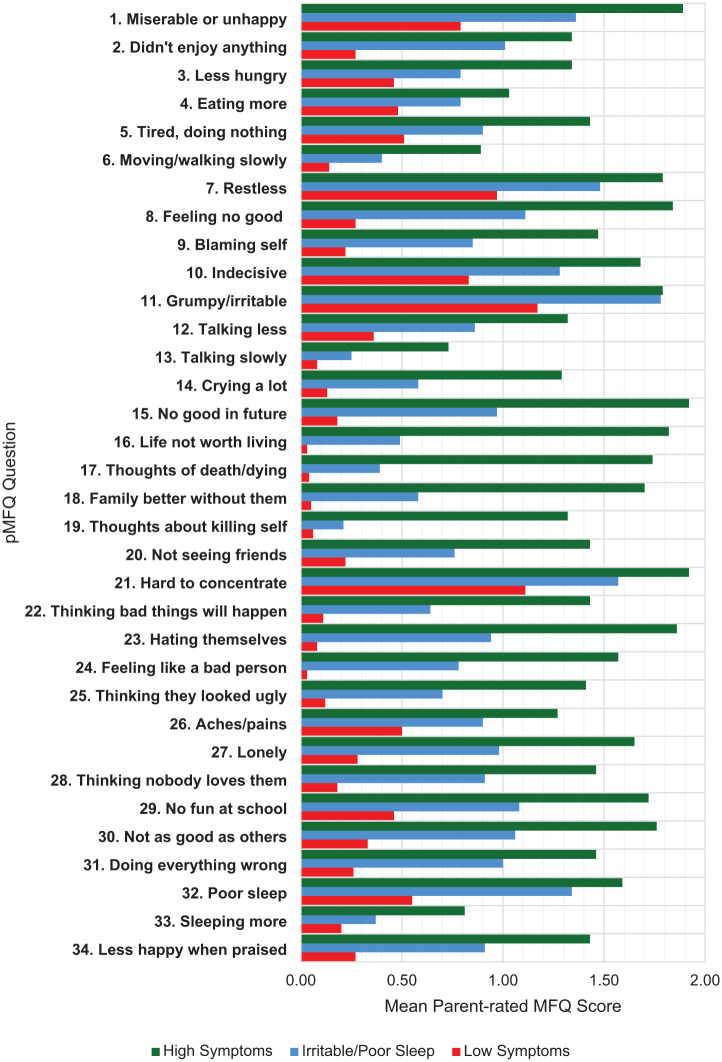
Mean pMFQ item scores for each latent profile. *Note*. pMFQ = parent-rated mood and feelings questionnaire.

Latent profile analysis of the 34 parent-rated depression symptoms from the MFQ identified a 3-class model as the best-fitting model (see Supplemental Material). Figure 1 shows the three distinct classes: ‘low symptoms’ (48.5%), ‘irritable/poor sleep’ (36.1%), and ‘high symptoms’ (15.5%).

In the low symptoms’ class, mean scores for 26 of the 34 items were low (mean = <0.5). Scores for symptoms relating to being miserable/unhappy (item 1), restlessness (item 7), indecisiveness (item 10), and poor sleep (item 32) were slightly elevated (mean = 0.5–1.0). Symptoms relating to irritability (item 11) and concentration (item 21) were scored somewhat high (mean = 1.0–1.5).

In contrast, in the high symptoms class all symptoms had a mean score above 0.5, with mean scores for 16 of the 34 items being high (mean = >1.5). Notably, symptoms for items relating to loss of hope for the future, feeling that life is not worth living, thoughts of death and dying, and that their family would be better off without them (items 15–18) were exceptionally high in this group (mean = 1.7–1.9). Thoughts of killing self was scored somewhat high (mean = 1.3) in this group. Mean scores for items relating to low self-esteem (e.g., items 23–24) were high for this group (mean = 1.6–1.9).

Finally, in the irritable/poor sleep class, mean scores for only 6 of the 34 items were low (mean = <0.5), mean scores for 16 items were slightly elevated (mean = 0.5–1.0), mean scores for eight items were somewhat high (mean = 1.0–1.5), and mean scores for two items relating to irritability (item 11) and inattention (item 21) were high (mean = >1.5). This class generally exhibited intermediate scores between the ‘Low symptoms’ and ‘High symptoms’ classes. However, scores for irritability (item 11, mean = 1.78) and poor sleep (item 32, mean = 1.34) exhibited mean scores that closely resembled those of the ‘High symptoms’ class, explaining the rationale behind the nomenclature of the ‘Irritable/poor sleep’ class.

### Associations With Identified Symptom Clusters

#### High Symptoms Class

As described in Table 1, our analysis found evidence that the presence of ODD (OR = 3.68, 95% CI [1.66, 8.15]) and CD (OR = 4.98, 95% CI [1.93, 12.85]) were associated with being in the high symptoms compared to low symptoms class. There was also evidence that higher ADHD-related impairment scores (OR = 1.17, 95% CI [1.04, 1.32]), estimated gross family income of less than £20,000 (OR = 3.62, 95% CI [1.37, 9.55]), and having a comorbid anxiety disorder (OR = 5.81, 95% CI [1.52, 22.26]) were associated with being the high symptoms class compared to the low symptoms class. Sex, ADHD subtypes, autism screening score, family history of depression, age, and IQ did not clearly differentiate the two classes.

**Table 1.** Associations of Latent Profiles With Selected Variables From Clinical and Impairment Data.

Clinical and demographic variables	Class 1: ‘low symptoms’	Class 2: ‘irritable/poor sleep’	Class 3: ‘high symptoms’	Class 2 vs. Class 1	Class 3 vs. Class 1
Sample size (n, % of sample)	118 (48.5)	90 (36.1)	38 (15.5)		
Total pMFQ score (mean, *SD*)	11.76 (0.53)	30.36 (0.66)	51.18 (1.27)	*OR* = 2.29 [95% CI 1.65, 3.19], *p* < .001, *q* < .005^a^	*OR* = 3.34 [95% CI 2.33, 4.80], *p* < .001, *q* < .005^a^
Female sex (%)	11.8	25.9	21.0	*OR* = 2.61 [95% CI 1.23, 5.52], *p* = .012, *q* = .029	*OR* = 1.99 [95% CI 0.76, 5.23], *p* = .160, *q* = .187
ADHD subtype (%)				*OR* = 0.69 [95% CI 0.41, 1.16], *p* = .164, *q* = .187	*OR* = 0.56 [95% CI 0.26, 1.23], *p* = .151, *q* = .187
*Combined*	82.8	89.3	90.9		
*Inattentive*	4.8	4.8	6.1		
*Hyperactive-Impulsive*	12.4	5.9	3.0		
Comorbidities (%)					
*Anxiety disorders*	3.4	10.5	17.2	*OR* = 3.29 [95% CI 0.95, 11.39], *p* = .061, *q* = .098	*OR* = 5.81 [95% CI 1.52, 22.26], *p* = .01, *q* = .027
*CD*	8.5	18.0	31.6	*OR* = 2.36 [95% CI 0.99, 5.60], *p* = .052, *q* = .096	*OR* = 4.98 [95% CI 1.93, 12.85], *p* = .001, *q* = .005
*ODD*	40.1	61.1	71.1	*OR* = 2.35 [95% CI 1.32, 4.20], *p* = .004, *q* = .016	*OR* = 3.68 [95% CI 1.66, 8.15], *p* = .001, *q* = .005
Autism Screening Score (mean, *SD*)	10.42 (0.51)	13.50 (0.78)	12.51 (1.08)	*OR* = 1.08 [95% CI 1.03 -1.14] *p* = .001, *q* = .005	*OR* = 1.06 [95% CI 0.997 -1.13], *p* = .059, *q* = .098
Family history of depression (%)	33.5	48.1	50.0	OR=1.83 [95% CI 1.03, 3.28], *p* = .041, *q* = .082	*OR* = 1.98 [95% CI 0.94, 4.18], *p* = .072, *q* = .102
Low Income (%)	53.6	67.9	80.7	*OR* = 1.84 [95% CI 0.96, 3.50], *p* = .065, *q* = .098	*OR* = 3.62 [95% CI 1.37, 9.55], *p* = .009, *q* = .027
Age (mean, *SD*)	9.14 (0.17)	8.88 (0.20)	9.11 (0.33)	*OR* = 0.93 [95% CI 0.80, 1.08], *p* = .343, *q* = .374	*OR* = 0.99 [95% CI 0.81, 1.22], *p* = .933, *q* = .933
ADHD Impairment (mean, *SD*)	18.34 (0.39)	19.49 (0.54)	20.64 (0.57)	*OR* = 1.07 [95% CI 0.98, 1.17], *p* = .145, *q* = .187	*OR* = 1.17 [95% CI 1.04, 1.32], *p* = .008, *q* = .027
IQ (mean, *SD*)	86.70 (1.11)	85.03(1.47)	81.14 (2.35)	*OR* = 0.99 [95% CI 0.97, 1.01], *p* = .376, *q* = .392	*OR* = 0.96 [9% CI 0.93, 0.999], *p* = .04, *q* = .082

*Note. q*, *p*-values adjusted for multiple testing (using false discovery rate; FDR). pMFQ = parent-rated mood and feelings questionnaire; CD = conduct disorder; ODD = oppositional defiant disorder.

a Statistical software reported p-value of 0.000 and further decimal places could not be viewed.

#### Irritable/Poor Sleep Class

Our analysis found evidence that higher autism screening score was associated with being in the irritable/poor sleep class compared to the low symptoms class (OR = 1.08, 95% CI [1.03, 1.14]). There was also evidence that oppositional defiant disorder (OR = 2.35, 95% CI [1.32, 4.20]) and female sex (OR = 2.61, 95% CI [1.23, 5.52]) was associated with the irritable/poor sleep class compared to the low symptoms class. Analyses did not find strong evidence of associations for ADHD subtype, anxiety disorders, conduct disorder, family history of depression, low estimated family income, age, ADHD-related impairment, or IQ.

## Discussion

This study aimed to investigate heterogeneity in the presentation of depression in young people with ADHD and to examine clinical and demographic correlates with each profile. We identified three distinct clusters of symptom profiles: ‘Low symptoms’ (which we used as our reference group), ‘Irritable/poor sleep’, and ‘High symptoms’. We found evidence of association of comorbid oppositional defiant disorder (ODD), conduct disorder (CD), ADHD-related impairment, low family income, and anxiety disorders with the high symptoms class. We found evidence of associations between higher autism symptom scores, ODD, and female sex and being in the irritable/poor sleep class.

### Presentation of Depression Symptoms in ADHD

Around half of the participants (48.5%) in this study fell into a class exhibiting relatively low depressive symptoms in comparison to the other classes, with high mean scores for symptoms of depression that are consistent with common symptoms of ADHD, including irritability and difficulty concentrating. In this low depressive symptom class, scores were also slightly elevated for restlessness, indecisiveness, being miserable or unhappy, poor sleep, and tiredness. Many of these items overlap phenotypically with typical features of ADHD, for example, difficulty concentrating and restlessness closely resemble inattention and hyperactivity – two core features of ADHD (Thapar & Cooper, 2016).

The features of the low symptoms class share similarities with those identified by Fraser et al. (2018), who found irritability, difficulties concentrating, and restlessness were the most commonly reported symptoms on the pMFQ. This is unsurprising given that Fraser et al. (2018) also studied the SAGE sample used in our study, and that the low symptoms class is the largest class and therefore likely to be most similar to the whole sample used by Fraser et al. (2018). Joseph et al. (2019) observed similar results, indicating irritability, difficulty concentrating, and indecisiveness as the most common depression symptoms in children with ADHD. Although existing studies have offered valuable contributions by identifying relative frequencies of reported symptoms in young people with ADHD, they are unable to show how these symptoms cluster together in different individuals. Our study contributes to this body of research by identifying a distinct depression symptom profile characterised by relatively low prevalence of depression symptoms, except for some depression symptoms that overlap with ADHD itself.

In addition to the low symptoms class, our study found two further distinct presentations of depression in the sample, the high symptoms class and the irritable/poor sleep class. The high symptoms class, representing 15.5% of the sample, showed very high depression symptoms across all items, notably featuring high rates of suicidal cognitions and low self-esteem. This is consistent with findings from Diler et al. (2007), who also identified suicidality and poor self-esteem as prevalent features of major depressive disorder (MDD) in young people with ADHD. The elevated suicidality symptom scores in our high symptoms class align with existing literature highlighting the increased suicide risk in ADHD populations (Biederman et al., 2008). However, our depression measure only captured suicidal cognitions, which does not necessarily translate to suicidal acts. Our findings contribute to our understanding of more severe presentations of depression in young people with ADHD. Whereas existing literature has isolated individual symptoms as potential discriminators of depression in ADHD, our study has identified symptoms that co-occur significantly within a subset of the sample that exhibit a more severe constellation of depression symptoms.

The remaining presentation of depression symptoms, the irritable/poor sleep class, represented 36.1% of the sample. This presentation exhibited overall depression symptom scores that were intermediate to the other two classes, with particularly high rates of irritability (being grumpy with parents) and poor sleep. This pattern of symptoms was unexpected and may be less easily explained than the other classes identified in our study. Although irritability is common in young people with ADHD (Eyre et al., 2019), its high mean score within this class suggests heterogeneity within the sample, with a subset of individuals showing particularly high levels of irritability. Our findings suggest that in this sample, irritability does not always co-occur alongside other depressive symptoms. This is consistent with findings from Diler et al. (2007), which found that high levels of irritability do not necessarily indicate MDD in young people with ADHD, as irritability is extremely common in those with ADHD without comorbid MDD. On the other hand, evidence from a longitudinal study suggests that children with ADHD who present with *persistent* irritability are at elevated risk of developing depression (Eyre et al., 2019). One important consideration is that our study only took a snapshot of symptoms over the last 2 weeks, and therefore, we cannot tell if levels of irritability were transient or persistent. If irritability were a precursor to depression, we might have expected the irritable/poor sleep class to be younger than the high symptoms class, but this was not the case in our study. Therefore, our findings support the need for further research on the relationship between irritability and depression in ADHD.

There are several potential explanations for the high prevalence of poor sleep in this class. It is well-documented that young people with ADHD commonly experience sleep problems; a meta-analysis found this population to be significantly more impaired across most sleep measures compared to the general population (Cortese et al., 2009). Furthermore, sleep problems in youth are associated with a range of mental health outcomes, including depressive symptoms, irrespective of ADHD status (Loram et al., 2023). Evidence also suggests that poor sleep precipitates irritability, which might explain their co-occurrence in this class, although a causal mechanism has not been established (Nelson et al., 2022). This unique and somewhat unexpected profile of depression symptoms may warrant further research as it may have distinct aetiological mechanisms and treatment strategies.

### Correlates of Different Presentations of Depression Symptoms in ADHD

Compared to our reference group, the low symptoms class, our analyses found evidence of association for the irritable/poor sleep class with high autism symptom scores, oppositional defiant disorder (ODD), and female sex. This suggests a potentially more neurodevelopmental picture for those young people with ADHD who present with this pattern of depression symptoms, and is consistent with the well-documented association of irritability with neurodevelopmental disorders including ADHD and ASD (Shaw et al., 2014; Simonoff et al., 2012). Furthermore, ASD and ADHD have both been found to be associated with higher rates of sleep disorders such as insomnia compared to people without ASD and ADHD (Cortese et al., 2009; Díaz-Román et al., 2018). For these reasons, it may be possible that the irritable/poor sleep presentation of depression in young people with ADHD might be explained by its association with autistic traits. Thus, one, explanation for this group is that high levels of irritability and poor sleep represent a particular presentation of depression. However, an alternative explanation is that this group are presenting with subthreshold depression symptoms (i.e., the intermediate levels of depression symptoms compared to the low and high groups), and that the high levels of irritability and poor sleep presented are more general symptoms associated with neurodevelopmental phenotypes, rather than being specific symptoms of depression. Further research, including investigation of longer-term outcomes, are needed to better understand this group.

Compared to the low symptoms class, we found evidence for associations with the high symptoms class for oppositional defiant disorder (ODD) and conduct disorder (CD). This potentially suggests a presentation more characterised by behavioural problems for this depression symptom class, where comorbidity with behavioural disorders appears to be more common. Findings from non-ADHD samples suggest that individuals with co-occurring behavioural disorder and depression experience worse outcomes compared to those with depression alone (Ingoldsby et al., 2006). There is evidence that ODD and CD are associated with increased suicidality and that having comorbid CD and depression increases the risk of suicide attempts compared to having either condition alone (Gatta et al., 2023). This documented association between behavioural disorders and suicidality is consistent with the characteristics of our high symptoms class, where suicidal cognitions are a key feature. Our study also found that rates of ODD increased incrementally between classes, suggesting that behavioural disorders may be a marker of the severity of depression symptoms.

In addition, we found evidence of associations for the high depression symptoms class with ADHD-related impairment, anxiety disorders, and low income. Depression and anxiety disorders commonly co-occur, and anxiety disorders are a well-established antecedent to depression (Kessler et al., 2005; Rice et al., 2017). Studies have also found that individuals with lower family income exhibit higher rates of depression and anxiety, as well as behavioural disorders such as ODD and CD (Yang et al., 2023). Likewise, children who are more impaired by their ADHD symptoms, particularly in familial and social domains, are more likely to develop depression (Capps et al., 2023).

While previous literature has identified many of these variables as distinct risk factors for depression, our findings contribute to the field by suggesting that young people with ADHD, alongside these risk factors, are more likely to present with a unique, more severe, and potentially more impairing constellation of depression symptoms, characterised by high suicidal cognitions, and poor self-esteem.

### Strengths and Limitations

Our study used a large clinical sample using rigorous measures for clinical and impairment data, including a thorough screening process for comorbid psychiatric disorders. However, findings should be considered in light of the study limitations. The sample is of entirely of British Caucasian origin, and therefore findings cannot be generalised to other ethnic groups. The fact that we used a clinical sample suggests the likelihood of increased severity of symptoms and higher occurrence of comorbidities (Woodward et al., 1997). Although this is very useful for research purposes, it may not be generalisable to community settings. The sample was also predominantly male, which is representative of other clinical samples of those with ADHD (Thapar & Cooper, 2016) and of the Welsh population of those with an ADHD diagnosis (Martin et al., 2024), but is less representative of population samples where the excess of affected males is less marked (Thapar & Cooper, 2016). This may have impacted our findings, given that depression symptoms may manifest differently according to gender (Thapar et al., 2022). The exclusion of individuals with comorbid ASD from our sample limits any conclusions that we can draw about the relationship between ADHD and autism and how depression symptoms present in individuals with comorbid ADHD and ASD, which may be particularly relevant to the irritable/poor sleep group. Future research into depression symptom heterogeneity in those meeting thresholds for ADHD in population samples, as well as in clinical samples of those with an ADHD diagnosis, may overcome some of these limitations. This study did not examine in detail treatment history of participants or side effects of pharmacological treatments that may contribute to their depression symptoms. However, none of the children in the SAGE sample were taking antidepressant medication according to parent report at baseline. A previous study in the same sample used here (SAGE) reported that 80% of participating children were receiving stimulant medication for their ADHD according to parent report at baseline (Eyre et al., 2019), consistent with what we might expect in a clinical sample, and this may therefore affect findings given that ADHD medication has been found to be associated with reduced risk of depression (Chang et al., 2016). It is also important to note that our depression symptoms measure is a screening tool and cannot be used to diagnose depressive disorders, although it can indicate who might be at increased risk. We used parent-ratings of depression symptoms because previous work using this measure in this sample suggests that adolescents with ADHD may under-report their own depression symptoms compared to their parent’s reports, while the opposite was observed in a population sample (Fraser et al., 2018). However, the use of parent – instead of self-reports may have impacted findings.

### Implications

While existing research has highlighted common depression symptoms in those with ADHD, the findings of our study provide new insights into (i) how specific depression symptoms cluster together into distinct symptom profiles in those with ADHD and (ii) how these profiles correlate with clinical and demographic traits. Our findings may be useful for clinicians in understanding how depression symptoms might present in various subgroups and to identify which young people with ADHD may be at higher risk of more severe presentations of depression symptoms.

Our findings suggest that clinicians working with young people with ADHD should be vigilant for symptoms such as suicidal cognitions and low self-esteem, which were indicators of a more severe depression symptom presentation in our sample of young people with ADHD. Our study also highlights the importance of recognition of comorbid behavioural disorders in young people with ADHD, as these were associated with a severe pattern of depression symptoms including higher suicidal cognitions. Our findings also suggest that girls and individuals with more autistic traits might be more likely to present with depression that is characterised by irritability and poor sleep symptoms.

## Conclusions

Our study highlights the heterogeneity of depression symptoms in young people with ADHD. We identified three distinct presentations of depression. Firstly, a low depression symptoms group representing around half of the sample. Secondly, a high symptoms group including high suicidality and low self-esteem symptoms, which was associated with behavioural disorders. Thirdly, a group characterised by high levels of irritability and poor sleep with intermediate levels of other depression symptoms, which was associated with higher autism symptoms. Our findings contribute to our understanding of which depression symptoms might cluster together into specific depression presentations in young people with ADHD, and the risk factors that are associated with these specific presentations of depressive symptoms.

## Supplemental Material

sj-docx-1-jad-10.1177_10870547251366783 – Supplemental material for Investigating the Symptom Presentation of Depression in Children With ADHD Supplemental material, sj-docx-1-jad-10.1177_10870547251366783 for Investigating the Symptom Presentation of Depression in Children With ADHD by Gareth Williams, Victoria Powell, Olga Eyre, Anita Thapar and Lucy Riglin in Journal of Attention Disorders
