# Supplementary material for: Investigating the Symptom Presentation of Depression in Children With ADHD
Source: J Atten Disord. 2025 Oct 2;30(4):449–59. doi: 10.1177/10870547251366783 (PMC12953668; doi:10.1177/10870547251366783)
Supplement: sj-docx-1-jad-10.1177_10870547251366783 – Supplemental material for Investigating the Symptom Presentation of Depression in Children With ADHD [file sj-docx-1-jad-10.1177_10870547251366783.docx]

**Supplementary Material**

**Selecting the number of profiles**

Latent profile analysis was used to derive profiles of the 34 depression items. Models were run with item variances constrained to be equal across classes and using 1000 random starting values and 100 optimizations (STARTS = 1000 100 in Mplus) (Muthén & Muthén, 1998-2012). Model fit significantly improved, as indicated by the fall in loglikelihood value, sample size adjusted Bayesian information criterion, Vuong-Lo-Mendell-Rubin Likelihood Ratio Test and Bootstrapped Likelihood Ratio Test, from the one- to three-class solution (see Supplementary Table 1). The four-class solution was unidentified as the number of people estimated to be in one of the classes did not exceed the number of variable means estimated for each class (34 depression items). The 3-class solutions was therefore selected.

| **Supplementary Table 1**. Model fit indices for latent profile analysis | | | | | | | | |
| --- | --- | --- | --- | --- | --- | --- | --- | --- |
| k | NFP | LL | BIC | ssaBIC | Smallest class | Entropy | VLMR-LRT  p-value | BLRT  p-value |
| 1 | 68 | -9061 | 18497 | 18281 | - | - | - | - |
| 2 | 103 | -7768 | 16102 | 15776 | 31% | 0.99 | <0.0001 | <0.0001 |
| 3^*^ | 138 | -7288 | 15336 | 14899 | 15% | 0.98 | 0.0011 | <0.0001 |
| 4^#^ | 173 | -7141 | 15234 | 14686 | 14% | 0.98 | 0.5741 | <0.0001 |
| k = number of classes, NFP = number of free parameters, LL = Loglikelihood, ssa = sample size adjusted, BIC = Bayesian Information Criteria, VLMR-LRT = Vuong-Lo-Mendell-Rubin Likelihood Ratio Rest; BLRT = Bootstrapped Likelihood Ratio Test. ^*^Final model ^#^Model unidentified. | | | | | | | | |
